# Glucocorticoids coordinate changes in gut microbiome composition in wild North American red squirrels

**DOI:** 10.1038/s41598-022-06359-5

**Published:** 2022-02-16

**Authors:** Lauren Petrullo, Tiantian Ren, Martin Wu, Rudy Boonstra, Rupert Palme, Stan Boutin, Andrew G. McAdam, Ben Dantzer

**Affiliations:** 1 Department of Psychology, University of Michigan, Ann Arbor, MI 48108 USA; 2 Department of Biology, University of Virginia, Charlottesville, VA 22904 USA; 3 Department of Biological Sciences, Centre for the Neurobiology of Stress, University of Toronto Scarborough, Toronto, ON M1C 1A6 Canada; 4 Unit of Physiology, Pathophysiology and Experimental Endocrinology, Department of Biomedical Sciences, University of Veterinary Medicine Vienna, Veterina ¨rplatz 1, 1210 Vienna, Austria; 5 Department of Biological Sciences, University of Alberta, Edmonton, AB T6G 2E9 Canada; 6 Department of Ecology and Evolutionary Biology, University of Colorado, Boulder, CO USA; 7 Department of Ecology and Evolutionary Biology, University of Michigan, Ann Arbor, MI 48108 USA

**Keywords:** Microbial communities, Microbial ecology, Ecology

## Abstract

The gut microbiome impacts host health and fitness, in part through the diversification of gut metabolic function and pathogen protection. Elevations in glucocorticoids (GCs) appear to reduce gut microbiome diversity in experimental studies, suggesting that a loss of microbial diversity may be a negative consequence of increased GCs. However, given that ecological factors like food availability and population density may independently influence both GCs and microbial diversity, understanding how these factors structure the GC-microbiome relationship is crucial to interpreting its significance in wild populations. Here, we used an ecological framework to investigate the relationship between GCs and gut microbiome diversity in wild North American red squirrels (*Tamiasciurus hudsonicus*). As expected, higher GCs predicted lower gut microbiome diversity and an increase in metabolic taxa. Surprisingly, but in line with prior empirical studies on wild animals, gastrointestinal pathogens decreased as GCs increased. Both dietary heterogeneity and an upcoming food pulse exhibited direct effects on gut microbiome diversity, whereas conspecific density and reproductive activity impacted diversity indirectly via changes in host GCs. Our results provide evidence of a gut–brain axis in wild red squirrels and highlight the importance of situating the GC-gut microbiome relationship within an ecological framework.

## Introduction

The intimate symbiosis between animals and their microbiomes has become a major area of focus for animal behavior, ecology, and evolution research over the last decade. In vertebrates, the gut microbiome interacts strongly with other host physiological systems^1^. Gut microbiota are sensitive to changes in host immune function^2^, brain development and behavior^3,4^, circadian rhythms^5^, and metabolism^6^. Beyond these effects, the gut microbiome also responds to the host endocrine system. In wild female primates, reproductive hormones like estrogen and progesterone are associated with differences in gut microbiome composition^7,8^. In humans, both androgens and estrogens, as well as metabolic hormones like insulin, are linked to variation in gut microbiota^9,10^. Such connections reflect a larger “gut–brain axis” through which the gut microbiota and nervous system communicate^11^.

Recently, glucocorticoids (GCs) have emerged as a central component of the bidirectional gut–brain axis^12^. GCs are metabolic hormones produced via the activation of the hypothalamic–pituitary–adrenal axis. They are involved in energy regulation and the physiological stress response^13^, and can induce adaptive phenotypic plasticity in response to environmental change^14^. For example, elevated GCs can enhance fitness by facilitating transitions between life history stages^15^, supporting the energetic demands of reproduction^16^, and improving survival in response to fluctuating temperatures and food availability^17^. GCs may also induce adaptive plasticity in the gut microbiome, a host microbial community that responds rapidly to changes in ecology. Shifts in gut microbiome composition can regulate energy balance as ambient temperatures rise and fall^18^, and can enhance digestion efficiency as an animal’s energetic demands increase (e.g., during reproduction) or as resource availability fluctuates^19–21^.

**Figure 1:**
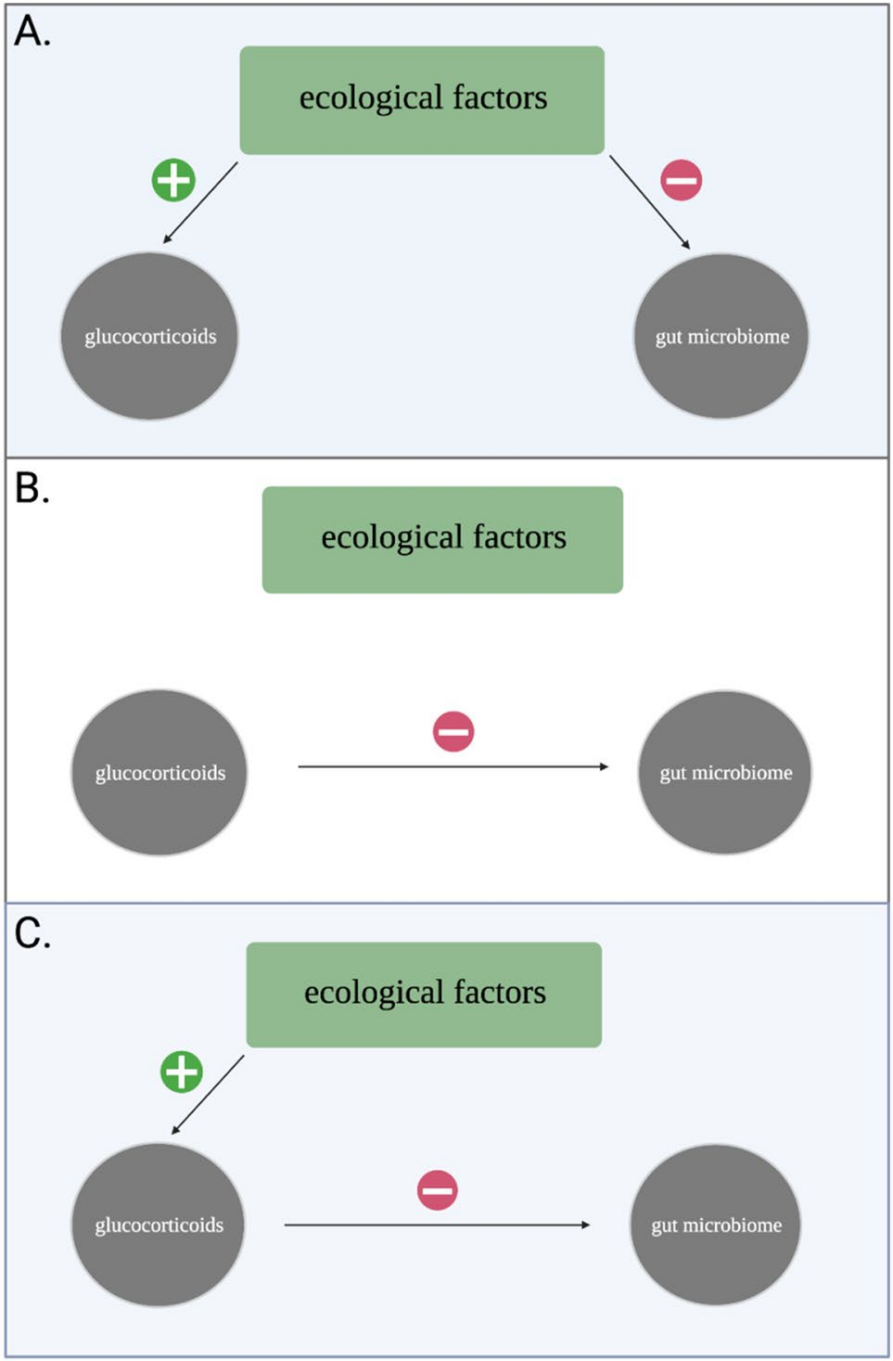
Conceptual model demonstrating how ecological factors structure the relationship between glucocorticoids and the gut microbiome. (**A**) Environmental covariance results in direct effects on both variables and diminishes a detectable effect of GCs on gut microbiome diversity. (**B**) Ecology does not influence either variable and a direct effect of GCs on gut microbiome diversity is preserved. (**C**) Ecological factors influence gut microbiome diversity indirectly via host GCs. Note that the three scenarios are not mutually exclusive, such that a combination of direct (**A**) and indirect (**B**) effects may result in the appearance or absence of a relationship between GCs and gut microbiome diversity.

One measure of gut microbiome composition—alpha diversity, or the taxonomic diversity within a single community—appears particularly sensitive to changes in host GCs. A taxonomically diverse microbiome confers community stability and resilience, whereas a loss of diversity is presumed to have detrimental consequences via increased host susceptibility to pathogenic infection^22,23^. Animal studies in which GCs have been experimentally elevated have documented reduced gut microbiome diversity in response to elevated GCs^24,25^, while studies in unmanipulated populations have found no relationship^26,27^. This inconsistency may indicate that the link between GCs and gut microbiome diversity is modified by ecological factors (Fig. 1), yet these are rarely included in such analyses (Table 1). For example, an increase in food availability can cause transient elevations in GCs if conspecific density also increases due to that elevation in food availability^28^. If elevated density results in more frequent social interactions, it may enhance microbiome diversity directly via increased microbial transmission among conspecifics^29^. Such environmental covariance may drive the absence of a relationship between GCs and microbiome diversity in unmanipulated populations (Fig. 1)^30–32^, necessitating an integrative approach to determine how GCs impact the gut microbiome in wild animals.

**Table 1:** Selected prior experimental and correlational studies on the relationship between glucocorticoids and gut microbiome alpha diversity in captive and wild vertebrates.

Study	Species	N	GCs	Effect	Ecological factors included?
Noguera et al. (2018)^24^	Gulls (*Larus michahellis*)	29	Experimental	↓ ɑ-diversity	No
Stothart et al. (2019)^26^	Gray squirrels (*Sciurus carolinensis*)	29	Natural	No effect	No
Uren Webster et al. (2020)^25^	Atlantic salmon (*Salmo salar*)	168	Experimental	↓ ɑ-diversity	No
Vlčková et al. (2018)^27^	Gorillas (*Gorilla gorilla gorilla*)	42	Natural	No effect	No

In this study, we test the hypothesis that ecological factors structure the effects of GCs on gut microbiome alpha diversity in wild North American red squirrels (*Tamiasciurus hudsonicus)* living in the Yukon, Canada. Red squirrels are highly territorial animals that experience dramatic shifts in food availability and population density as a result of fluctuations in their preferred food source, seeds from white spruce trees (*Picea glauca*)^33^. Squirrels incorporate other food sources into their diet when seasonally available (e.g., fungi, bark, leaves, flowers)^34^, resulting in changes in dietary diversity that may directly impact gut microbiome diversity. However, spruce seeds comprise the majority of their diet^34^ despite their episodic availability. Masting events occur every 4–6 years in white spruce, resulting in the production of a superabundance of cones containing seeds that become available in the autumn. By contrast, few to no cones are available in non-mast years^33,35^. In anticipation of an upcoming spruce mast, squirrels exhibit an extended breeding season and concomitant behavioral changes: territoriality breaks down and conspecific interactions increase due to increased breeding frequency and infanticidal behavior^36,37^. An upcoming spruce mast may thus exert direct positive effects on gut microbiome diversity via more frequent social interactions, which leads to greater horizontal microbial transmission^29^.

Squirrel densities also fluctuate in parallel with food pulses, with densities at their lowest in the months prior to a mast and highest in the spring following a mast^28,38^. Although sociality is expected to increase gut microbiome diversity in group-living animals^29^, this effect may not occur in territorial species^39^. For example, elevated conspecific densities result in increased frequency of long-range territorial vocalizations emitted by red squirrels in our study population^40^, which can in turn reduce interaction frequency by deterring territorial intrusions^39,41^. Indeed, conspecific interactions in squirrels do not appear to vary with density, and the number of territorial intruders has been both negatively correlated^40^ and unrelated^41^ to density. Given that both actual and perceived increases in density cause GC elevations in red squirrels^28,42^, density may have indirect rather than direct effects on microbiome diversity due to the psychosocial stress of anticipating greater competition^28^.

We predicted to find an overall negative association between host GCs and gut microbiome alpha diversity, along with an increase in pathogenic taxa and taxa involved in host metabolism with increasing GCs. Similar to prior studies in wild animals (Table 1), we focused here on the unidirectional effects of GCs on microbial diversity to understand gut–brain axis function in the absence of an experimental manipulation, and because previously documented sensitivity of GCs to intrinsic and extrinsic factors in our population^28^ suggests it may mediate downstream effects on the microbiome. We used a multivariate structural equation modeling approach^43,44^ to integrate GCs, the gut microbiome, and exogenous ecological and host biological variables into a single causal network^45^. We tested a set of a priori hypothesized relationships related to the direct and indirect effects of dietary heterogeneity, an upcoming spruce mast, and conspecific density on both GCs and gut microbiome diversity (Fig. S1). We expected to find that dietary heterogeneity and an upcoming spruce mast would have direct positive effects on gut microbiome diversity. Conversely, we predicted that density would have an indirect negative effect on diversity by way of GC elevations. We additionally included biological factors (reproductive activity, sex, age) in our analysis, given their potential effects on GCs and microbiome composition^46–48^. We predicted that reproductive activity had positive direct effects on both GCs and gut microbiome diversity, as a result of increased energetic demands^49,50^ and conspecific interactions, respectively. We also expected that older age would predict lower microbiome diversity, and that males would exhibit greater microbiome diversity due to travel across territories for multiple mating in the breeding season^51^.

## Results

### Gut microbiome diversity is negatively associated with glucocorticoids

**Figure 2:**
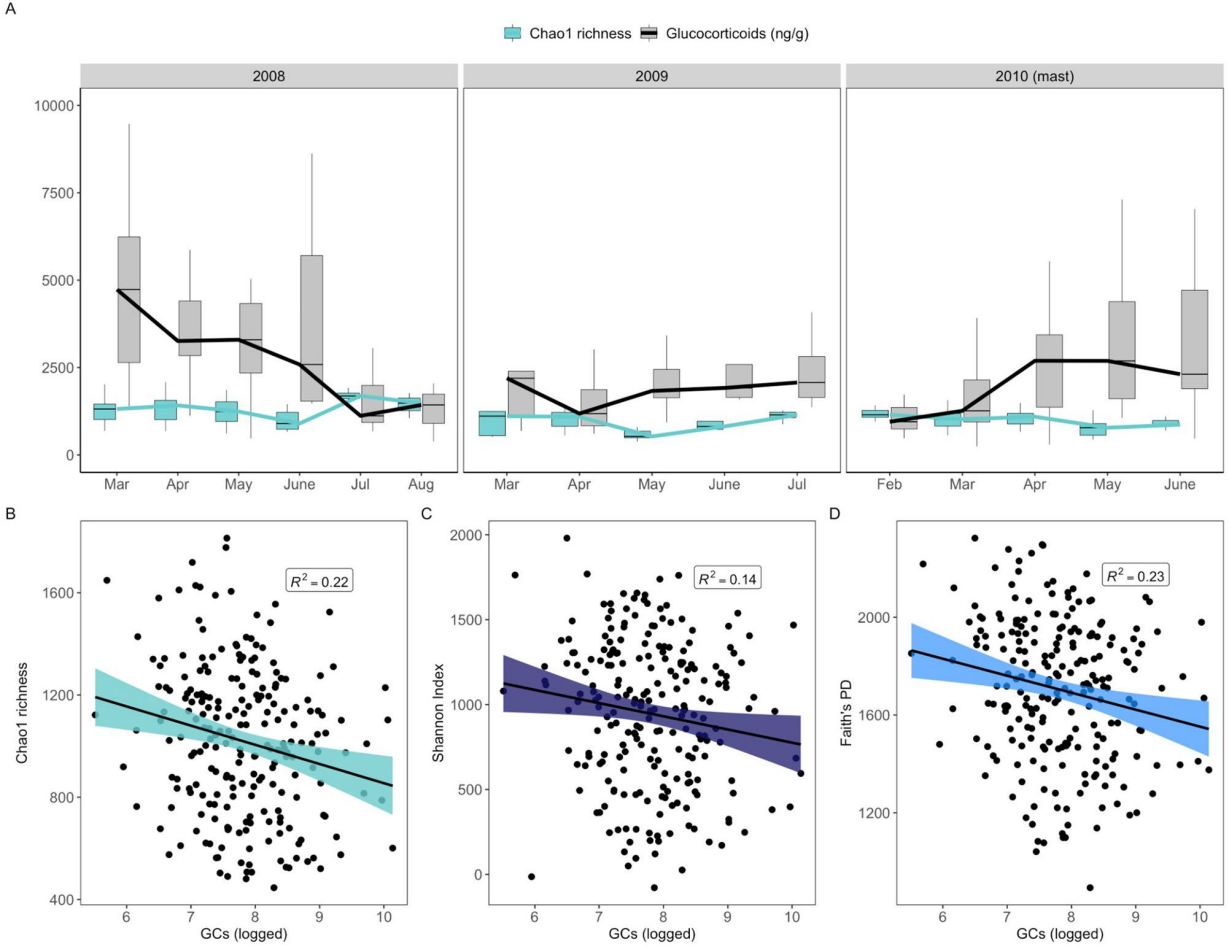
Host production of glucocorticoids predicts gut microbiome alpha diversity. (**A**) Boxplot and line graph showing the opposing relationship between mean fecal glucocorticoid metabolites (GCs, scaled) and median gut microbiome diversity (Chao1 richness) across each month of the three sampling years. Outliers removed from plot for visualization purposes. (**B**) Partial residual plots (points represent individual samples) showing the relationship between gut microbiome taxonomic richness (Chao1), (**C**) taxonomic richness and evenness (Shannon Index), and (**D**) phylogenetic diversity (Faith’s PD) and matched fecal glucocorticoid concentrations (GCs) (N = 227). R^2^ values (i.e., coefficients of determination) were obtained from linear mixed-effect models.

Both gut microbiome alpha diversity and GCs were highly variable across seasons in each of our sampling years, with gut microbial diversity reaching its maxima during the summer months of July and August (Fig. 2A), coinciding with increased dietary diversity^34,52^. GCs were highest in early spring (March), with the exception of the mast year of 2010 in which GCs steadily increased across the first part of the year (Fig. 2A). Consistent with our predictions and in line with prior studies in which GCs were experimentally manipulated (Table 1), GCs were negatively associated with gut microbiome alpha diversity. Individuals with greater GC concentrations exhibited relatively lower taxonomic diversity (i.e., species richness, Chao1: β = − 75.05 ± 25.91, t = − 2.90, P = 0.004; Fig. 2B). Greater GCs were also associated with lower Shannon Indices, a composite measure of species richness and evenness (Shannon: β = − 77.64 ± 36.00, t = − 2.16, P = 0.03, Fig. 2C), as well as lower phylogenetic diversity in the gut microbial community (Faith’s PD: β = − 66.97 ± 26.71, t = − 2.51, P = 0.01, Fig. 2D). The negative relationship between GCs and gut microbiome alpha diversity was robust to individual variation in GC production, with higher individually-averaged GCs similarly predicting lower species richness (β ± SE: − 0.027 ± 0.011, t = − 2.45, P = 0.02).

### Glucocorticoids predict changes in gut microbial taxa

**Figure 3:**
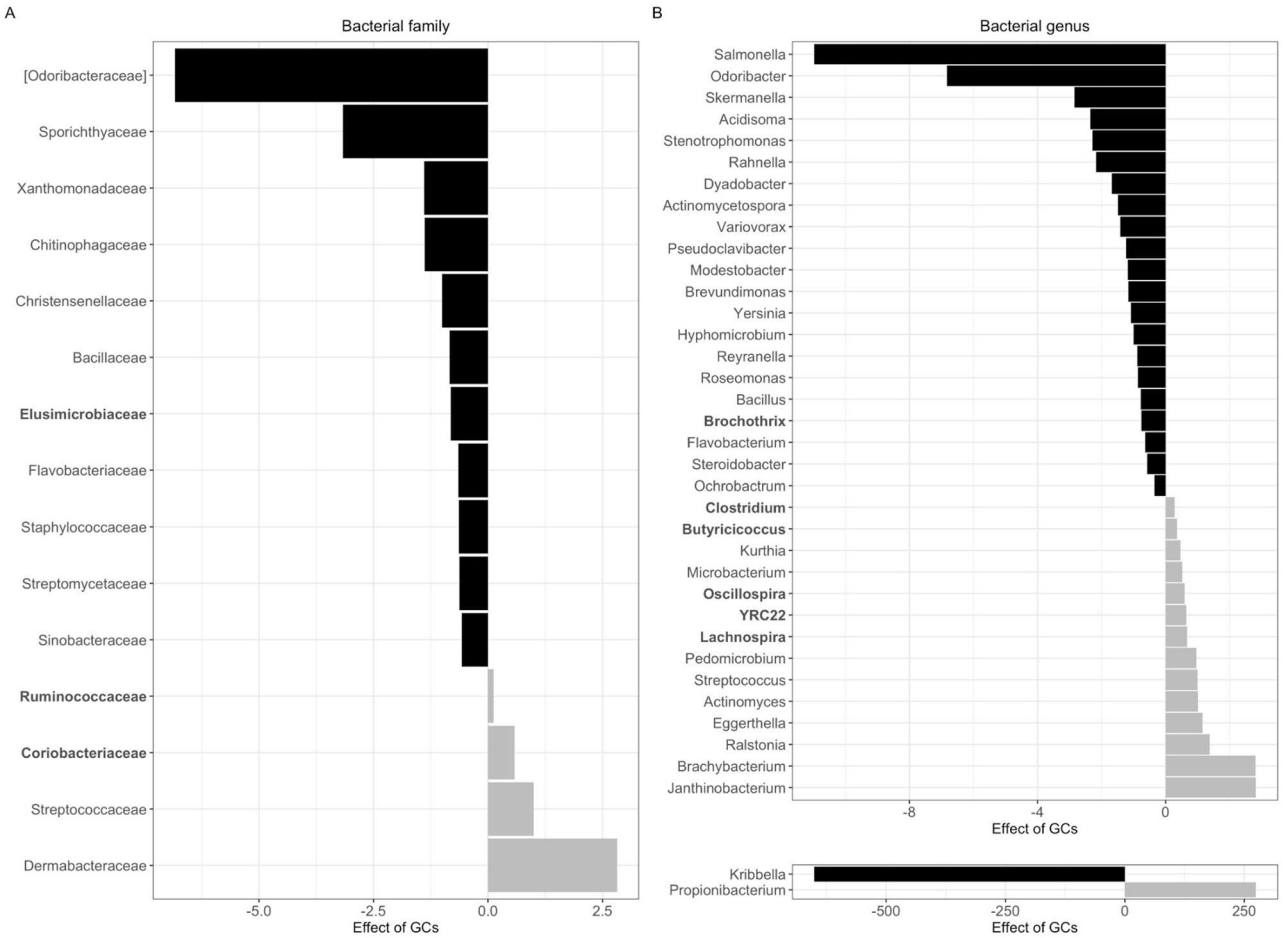
Gut microbial taxa that shift with increasing host glucocorticoids. Barplots depict bacterial families (**A**) and genera (**B**) whose relative abundance was significantly (Benjamini–Hochberg adjusted P < 0.05) predicted by changes in host glucocorticoid concentrations. Bold taxa exhibited a mean relative abundance > 0.01%. Effects of GCs reflect model estimates generated by negative binomial mixed models testing the effect of GCs on the relative abundance of each bacterial taxa, controlling for collection date, food supplementation status, and individual ID. Black bars represent a decrease in relative abundance with increasing GCs; grey bars represent an increase with increasing GCs. Taxa depicted at the bottom of Panel B (*Kribbella, Propionibacterium*) exhibited model estimates ~ 10× larger than the rest of the taxa and are therefore separated from the main plot for visualization purposes.

We constructed a series of negative binomial linear mixed-effects models to determine how reduced gut microbiome alpha diversity was reflected in changes at the taxonomic level and identify taxa whose relative abundances changed with increasing GCs. We found that elevated GCs were associated with changes in gut microbiome composition at both the family (Fig. 3A) and genus (Fig. 3B) levels. Increased GCs predicted shifts in the relative abundances of 15 bacterial families, predominantly decreases in rare bacterial families (i.e., taxa that contribute < 0.01% relative abundance to the microbial community) (Fig. 3A; Table S1). An exception was a reduction in *Elusimicrobiaceae*, which contributed an average of 0.13% relative abundance to the gut microbiome community (β = − 0.81, P_FDR_ < 0.0001). By contrast, elevated GCs were associated with an increase in *Coriobacteriaceae* (β = 0.57, P_FDR_ = 0.007)*, Streptococcaceae* (β = 1.0, P_FDR_ < 0.0001)*,* *Dermabacteraceae* (β = 2.81, P_FDR_ < 0.0001)*,* and *Ruminococcaceae* (β = 0.12, P_FDR_ = 0.02) (Fig. 3A). *Ruminococcaceae*, a family of largely cellulolytic and fibrolytic bacteria, is an abundant (~ 25% relative abundance) and core taxa in the red squirrel gut microbiome^52^.

**Figure 4:**
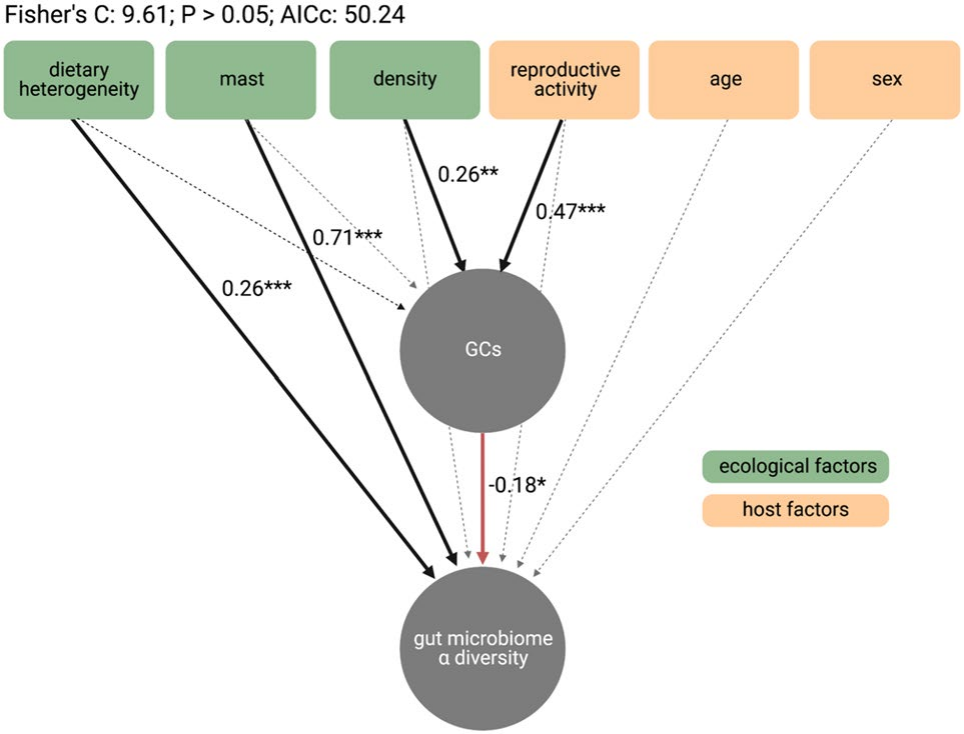
Ecology and host biology influence gut microbiome alpha diversity via changes in host glucocorticoids. Structural equation model assessing direct and indirect effects of ecological and host factors on glucocorticoids (GCs) and gut microbiome alpha diversity (Chao1 richness). Solid black arrows represent significant positive paths; solid red arrows represent significant negative paths; dotted arrows represent non-significant paths. Text labels indicate standardized beta estimates (i.e., effect sizes) and significance (P < 0.05*, P < 0.01**, P < 0.001***) for each of the predicted pathways tested in the SEM.

At the genus level, the relative abundances of 22 bacterial genera were significantly reduced with increasing host GCs. Similar to changes at the family level, the majority of bacterial genera reductions were rare taxa (Fig. 3B) with the exception of *Brochothrix* (mean 0.02% relative abundance). Contrary to our predictions but in line with a prior study on birds^24^, we found that two potentially pathogenic genera—*Yersinia* (β = − 1.09, P_FDR_ = 0.004)^53^ and *Salmonella* (β = − 10.98, P_FDR_ < 0.0001)^54^—decreased in relative abundance with increasing host GCs. Conversely, a greater proportion of abundant taxa were found to increase with increasing GCs (Fig. 3B). Greater host GCs predicted greater relative abundances of *Clostridium* (β = 0.28, P_FDR_ = 0.008)*, Butyricicoccus* (β = 0.35, P_FDR_ = 0.004)*, Oscillospira* (β = 0.60, P_FDR_ < 0.0001)*,* YRC22 (β = 0.65, P_FDR_ = 0.004)*, and Lachnospira* (β = 0.67, P_FDR_ = 0.02).

### The gut-brain axis mediates ecological effects on the gut microbiota

To determine how ecological and host factors contributed to the effects of GCs on gut microbiome alpha diversity, we fit a structural equation model (SEM) based on a set of a priori hypothesized pathways (Fig. S1). The SEM was constructed to test the relative direct and indirect effects of three ecological factors (dietary heterogeneity, an upcoming spruce mast, and conspecific density) and three host factors (reproductive activity, age, and sex) on GCs and gut microbiome diversity. Overall, the SEM revealed direct and indirect pathways by which ecological and host factors exert cascading effects on microbial diversity (Figure 4). In line with our predictions, dietary heterogeneity and the presence of an upcoming spruce mast both exhibited direct effects on gut microbiome diversity, such that a more heterogeneous diet (standardized β = 0.26, P < 0.001) and an upcoming spruce masting event (standardized β = 0.71, P < 0.001) led to greater microbial diversity. Tests of directed separation revealed that there was no effect of either dietary heterogeneity or an upcoming spruce mast on host GCs. By contrast, conspecific density and reproductive activity indirectly, but not directly, affected gut microbiome diversity via changes to host GCs. Higher squirrel densities (standardized β = 0.26, P = 0.006) and reproductive activity (standardized β = 0.47, P < 0.001) predicted greater GC concentrations, which in turn reduced microbial diversity (standardized β = − 0.18, P = 0.01). There was no effect of age or sex on gut microbiome diversity, and tests of directed separation similarly found no effect of age or sex on host GCs.

## Discussion

Determining if ecological factors structure the relationship between glucocorticoids (GCs) and gut microbiome alpha diversity is crucial to interpreting the adaptive value of the GC-microbiome connection in wild animals. Here, we demonstrate that the link between GCs and gut microbiome alpha diversity is robust to ecological factors that directly influence the gut microbiome in wild populations. Our findings additionally suggest that GCs can integrate changes in ecology and host biology to induce plasticity in the gut microbiome.

Gut microbiome alpha diversity varied across seasons and peaked in summer, coinciding with the period of greatest dietary heterogeneity^34,52^ (Fig. 2A). GCs exhibited greater variability overall: GCs reached their peak in the early spring in non-mast years (2008, 2009), but in summer of the mast year (2010) (Fig. 2A). Despite this variability, there was a significant negative association between GCs and gut microbiome diversity such that individuals with greater GCs exhibited lower alpha diversity across three separate measures (species richness, evenness, and phylogenetic diversity) (Fig. 2B–D). Though prior studies in unmanipulated populations did not detect a relationship between GCs and gut microbiome alpha diversity (Table 1), but see^55^ for oral microbiome), our results align with experimental studies in which GCs were manipulated. This suggests that gut–brain axis communication, particularly between GCs and gut microbiome composition, is under strong selection in our population.

As GCs increased, the taxa that decreased in the gut microbiome were overwhelmingly rare taxa that contributed < 0.01% in relative abundance to the overall community (e.g., *Odoribacteriaceae, Sporichthyaceae*) (Fig. 3). This finding aligns with expectations about the effects of disturbances on gut microbiome composition from an ecological perspective^56^. Resilience to microbial disturbances is greater among abundant taxa than non-abundant taxa^57^, likely due to divergent patterns of colonization and succession. Abundant taxa retain their position in microbial communities via selective filtering and by occupying core niches^56^. By contrast, rare taxa are incorporated into the microbiome largely via stochastic processes, though they contribute significantly to community alpha diversity measures^58^. A reduction in rare taxa with increasing GCs in our population may therefore indicate that the effects of elevated GCs on rare bacteria in the gut mimic those expected by broader microbial disturbances (e.g., antibiotics, infection)^59^.

A decrease in the relative abundance of rare bacteria may also serve to reorganize host metabolic priorities through replacement by core taxa that can better support changes in host energetic demands. Overall, we found that increases in host GCs were accompanied by increases in bacteria involved in host metabolism (Fig. 3). Both *Oscillospira,* which correlates with the consumption of spruce buds in the late spring^52^, and *Coriobacteriaceae,* a common rodent gut microbe involved in energy metabolism^60^, increased in relative abundance with increasing GCs. In experimental settings, housing stress caused an increase in *Coriobacteriaceae*^61^, suggesting that it may similarly contribute to maintaining energy balance in wild rodents facing challenging environmental conditions. We additionally found that individuals with elevated GCs exhibited greater relative abundances of *Ruminococcaceae,* a bacterial family of cellulolytic and fibrolytic bacteria involved in dietary acclimation in wild animals^20,62^. Together, our results suggest that in our study population, GCs may coordinate adaptive shifts in gut microbiome composition in response to increased energetic demands, seasonal changes in diet, or both.

Resistance to pathogens has been proposed as one of the major evolutionary advantages conferred by host microbial communities^63^ and butyrates, compounds produced via fermentation by microbiota in the large intestine^64^*,* are particularly critical to preventing intestinal pathogen invasion^65^. We found that the butyrate-producing bacteria *Butyriciococcus* (family *Ruminococcaceae)* and *Clostridium* were elevated in the red squirrel gut microbiome as GCs increased (Fig. 3). In line with this finding, elevated GCs were associated with lower relative abundances of two potentially pathogenic genera: *Salmonella* and *Yersinia*. *Salmonella* are rod-shaped, Gram-negative bacteria that can cause gastroenteritis in both rodents and humans upon infection^66^. Similarly, *Yersinia* (including *Y. pestis, Y. pseudotuberculosis,* and *Y. enterocolitica)* are commonly harbored in the gut microbiota of wild rodents and can lead to enteric and systemic disease^54,67^, though we note there is currently no evidence of *Yersinia* disease in our population. While a loss of these taxa with increasing GCs contradicts theoretical expectations of pathogen susceptibility as microbial diversity decreases, our findings align with a prior study in free-living birds in which elevated GCs similarly reduced the relative abundance of intestinal pathogens^24^, and in piglets in which GCs reduced *Salmonella*, specifically^68^. These data suggest that elevations in GCs may confer short-term protection against intestinal pathogens, potentially through transient increases in gut immune function or butyrate production. Confirmation of the pathogenicity of these taxa, however, requires high-resolution, strain-level genomic data outside the scope of the present study.

To disentangle the effects of ecological and host factors on the relationship between GCs and the gut microbiome, we constructed a structural equation model (SEM) based on a set of causal a priori hypothesized pathways (Fig. S1)^43,69^. As predicted, an upcoming spruce mast had a direct positive effect on gut microbiome alpha diversity, and this path was the strongest path in the SEM (Fig. 4). An increase in gut microbiome diversity in the mast year compared to the non-mast years aligns with our expectation of territorial breakdown and increased social interactions due to an extended breeding season in the months leading up to a masting event^36,37^. Given that the positive link between sociality and gut microbiome diversity is well-supported at least in some taxa^8,29,70^, squirrels may exhibit increased gut microbiome diversity due to greater horizontal transmission of microbes between conspecifics as social interactions become more frequent.

Similar to the effects of an upcoming spruce mast, dietary heterogeneity had a direct positive effect on microbial diversity, though this effect was approximately 2.5× weaker than that of the upcoming mast (standardized β = 0.26). Gut microbiome alpha diversity was greatest in the months in which the food available to red squirrels was most heterogeneous (e.g., fungi, buds, and seeds)^34^. Indeed, a varied diet is expected to increase microbiome diversity through greater substrate selection for diverse ecological niches^71^. This effect of dietary heterogeneity on gut microbiome diversity, coupled with prior work on the relationship between diet and gut microbiome composition in this population^52^, indicates that the red squirrel gut microbiome responds rapidly to shifts in food availability.

By contrast, conspecific density indirectly, but not directly, impacted gut microbiome diversity by way of GCs, lending support to previous findings that the frequency of social interactions (and thus horizontal transmission) is not related to squirrel density in this population^40^. In line with our expectations, elevated conspecific densities predicted increased host production of GCs^28^, which in turn predicted reduced gut microbiome diversity (Fig. 4). Red squirrels are highly sensitive to changes in density, and signals of both actual and perceived elevated density lead to GC increases independent of other ecological factors that covary with it (e.g., food)^28^. That elevated density reduced gut microbiome diversity via increasing GCs aligns with our understanding of the regulation of the gut–brain axis by the social environment, stress, and psychological state in laboratory rodents^72^. In the wild, vocalizations can buffer individuals from physical interactions with conspecifics even in times of high densities in highly territorial animals like red squirrels. Our results suggest that the indirect effects of increased density on gut microbiome diversity likely reflect the psychosocial stress of increased competition, demonstrating a novel link between social stress and the gut–brain axis in a wild mammal.

Reproductive state was the only host factor to exhibit an effect on gut microbiome diversity, and the effect was indirect and the strongest path to GCs in the model. As predicted, being reproductively active (e.g., scrotal for males, and breeding, gestating, or lactating for females) predicted greater GCs, and in turn a reduction in microbial diversity. In both males and females, reproduction increases host metabolic demands broadly^50^and, in females in our population, GCs specifically^49^. A consequent reduction in microbiome diversity may therefore better support host energy balance by increasing the relative abundance of core microbiota at the expense of rare taxa that contribute less to the metabolic functions of the community. Contrary to our predictions, we found no effect of sex or age on microbial diversity, and the SEM did not identify an effect of either on GCs via tests of directed separation^69^. Studies in humans and other mammals have found mixed effects of age on both GCs and microbial diversity^48,73,74^. Sex effects on microbiome composition related to hormones and behavior have been documented in experimental rodent models^47,75^, but studies in wild populations have not typically found sex differences in gut microbiome composition^76^.

A number of potential pathways may explain how host GCs impact gut microbiota. First, GCs can alter lipid metabolism, leading to lipid accumulation in the gut^77^. Increased lipid metabolism reduces taxonomic diversity in the gut microbiome of laboratory rodents as some bacteria exhibit sensitivity to lipid accumulation^78^. Second, elevated GCs can disrupt host circadian rhythms in laboratory settings^78^, reducing gut microbiome alpha diversity^79^. Finally, increased GCs can decrease mucin synthesis, which is integral to the stability of the gut microbiome and largely determines its composition^80^. Impaired mucin synthesis as a result of elevated GCs may therefore disrupt gut microbiome stability by reducing the resilience of the mucosal layer, leading to a reduction in non-core bacteria and an overall loss of community diversity^81^.

Of note is the bidirectionality of the gut–brain axis demonstrated in laboratory rodent studies^82,83^, and thus the potential bidirectionality of the GC-microbiome relationship in wild animals. Here, we focused on the unidirectional effects of GCs on gut microbiome diversity similar to prior studies (Table 1). However, gut microbiota can themselves regulate the hypothalamic–pituitary–adrenal axis, such that shifts in gut microbiome composition may directly modulate host production of GCs^84^ and contribute to a feedback loop between the two systems^85^. Statistical constraints inherent to structural equation modeling prevented us from incorporating a bidirectional relationship between GCs and microbial diversity into this study^69^. Nonetheless, the bidirectionality of the gut–brain axis has important implications for the evolution of the relationship between GCs and microbial diversity in wild mammals^11^. We encourage future research on wild populations to implement experimental frameworks whenever possible to better characterize the complexity of the GC-gut microbiome relationship.

## Methods

### Ethics statement

All methods were carried out in accordance with relevant guidelines and regulations. All research methods and protocols were conducted under animal ethics approvals from Michigan State University (AUF#04/08-046-00), University of Guelph (AUP#09R006), and University of Michigan (PRO00005866). All authors complied with the ARRIVE guidelines.

### Study population

Subjects for this study were wild North American red squirrels (*Tamiasciurus hudsonicus)* inhabiting a natural environment in southwest Yukon, Canada (61° N, 138° W). All subjects were continuously monitored as part of the Kluane Red Squirrel Project, a long-term field study that has been conducting a combination of live-trapping, focal behavioral observations, sampling, and experimental manipulations in the area since 1987^38,86^. Individual squirrels are marked with small metal ear tags and unique combinations of colored wire threaded through the ear tags. Individuals are monitored from birth to death in each year of study, roughly from March to October, using live-trapping and behavioral observations^38^. Individuals included in our study lived on one of three grids (Agnes or AG, Kloo or KL, Sulphur or SU). On AG, individuals were supplemented with peanut butter from October to May in each year for a separate experiment focused on experimentally increasing squirrel population density^28,38^. On KL and SU, no food supplementation was provided. All models controlled for food supplementation status given its potential impacts on gut microbiome composition.

### Sampling

We collected 227 samples from 88 individuals across three years (2008–2010). When individuals were captured, they were handled such that their unique identity could be determined (by reading ear tags), sexed, and their reproductive condition could be recorded. Fecal samples were collected opportunistically during live-trapping from underneath the traps using forceps. Following capture and handling of squirrels, fresh fecal samples were collected from underneath the traps, kept on wet ice until they could be frozen at − 20 °C within 5 h of collection in the field. Samples contaminated with urine were not collected and all samples were kept at − 20 °C until analysis. We removed one fecal pellet from each sample using sterilized forceps for microbiome sequencing and then used the rest of the sample to measure fecal GC metabolites.

### Age

The age of each squirrel was known as individual squirrels were uniquely tagged in their natal nest when they were ~ 25 days of age and age is accordingly recorded at each trapping event^38^.

### Reproductive activity

Squirrels were live-trapped regularly and handled using visualization to determine reproduction state. During each trapping event simultaneous with the collection of microbiome and hormone samples, the reproductive state of the individual was determined via abdominal palpation^86^. Males were considered reproductively active if their testes were scrotal, and not reproductively active if testes were abdominal. For females, pregnancy status was assessed via abdominal palpation for fetuses as well as by examining nipple condition. Females were determined to be reproductively active if they were gestating, lactating, or breeding based on nipple condition. We have previously found that females that are reproductively active (pregnant or lactating) have higher fecal GC metabolites than those that were not reproductively active whereas there were no differences in the effects of reproductive activity (presence or absence of scrotal testes) in males^49^.

### Dietary heterogeneity

Although red squirrels consume primarily white spruce seeds, they also consume a number of other foods (e.g., spruce bark and needles, willow leaves and buds, fungi, and bearberry flowers) and thus experience varying levels of seasonal dietary heterogeneity^34^. We coded dietary heterogeneity by ranking the availability of these different foods across seasons from greatest (3) to least (1). Samples collected prior to June of each year were ranked as 1, while samples collected in the month of June and late summer (July–August) were ranked as 2 and 3, respectively.

### Conspecific density

Densities (expressed as squirrels per hectare) for each grid of the study (KL, SU, AG) were calculated separately for each year (2008, 2009, 2010) across the dataset using census data. In May of each year, we determined the number of squirrels owning a territory on our study areas using a combination of live-trapping and behavioral observations. Because squirrels are diurnal, regularly exhibit territorial calls, and their territories are visually conspicuous, we were able to completely enumerate all squirrels living in our study areas.

### Sequencing and bioinformatics

Microbiome data used in this study are a subset of previously published data^52^. DNA extraction and sequencing was performed as described in Ren et al.^52^. Briefly, the V1–V3 hypervariable region of the 16S rRNA bacterial gene was amplified using two universal primers: 27F (5′-ARGGTTTGATCMTGGCTCAG-3′) and 534R (5′-TTACCGCGGCTGCTGGCAC-3′). Samples were barcoded for PCR amplification, pooled, gel purified, and then sequenced on an Illumina MiSeq using 300 bp paired-end sequences. Sequences were then filtered, quality controlled, and reads were successfully merged using QIIME^87^. Chimeras were removed using USEARCH^88^ and sequences determined to be non-chimeric by both de novo and reference-based algorithms were retained. Reads were clustered to OTUs using UCLUST^89^ with an identity threshold of 97% (genus-level). Mitochondria and chloroplast were removed, and samples were rarefied to 4000 reads per sample.

### Hormone metabolite analysis

The time period from collection in the field to freezing (~ 5 h) did not impact fecal GC metabolites^49^. We measured fecal GC metabolites using previously validated protocols^49,90^. Briefly, samples were lyophilized for 14–16 h, bathed in liquid nitrogen, and pulverized using a mortar and pestle. A subsample (0.05 g) was then extracted using 80% methanol where the samples were vortexed at 1450 RPM for 30 min followed by centrifuging for 15 min at 2500*g*^49^. The supernatant was then used in an enzyme-immunoassay that employed an antibody that measures GC metabolites with a 5α-3β,11β-diol structure^91^. We have previously validated this assay and shown that the antibody can accurately measure increases in adrenal production of GCs^49^. We have also shown that our measures of fecal GC metabolites are comparable across assays^92^. Using pooled samples that were run repeatedly on different plates (n = 115) in our laboratory show that the estimates of optical density for these pooled samples were highly repeatable (*R* = 0.851, 95% CI 0.543–0.925). Using a linear mixed-effects model, we partitioned the variance in the optical density recorded for the pooled samples that were run across these different plates and found that most of the variance was due to the sample itself (85.1%) with little of it being explained by intra-assay variation as all samples were run in duplicate (4.9%) or by inter-assay variation (9.9%).

### Statistical analysis

All statistical analyses were conducted in R (v. 3.5.2.) (R. Core Team, 2015). OTU and taxonomy tables were imported into R and merged into a phyloseq object for downstream analyses using the ape^93^ and phyloseq^94^ packages. All figures were created in R, with the exception of the conceptual model (Fig. 1) and the structural equation model figures (Figs. 4 and Fig. S1), which were created in bioRender (www.biorender.com).

### Alpha diversity

The estimate_richness() function in the phyloseq package was used to calculate the observed richness (Chao1) and Shannon Index of alpha diversity. Faith’s Phylogenetic Distances were calculated using the pd() function on the phyloseq object in picante^95^. Linear mixed-effects models were used to assess the relationship between bacterial diversity and fecal glucocorticoid metabolites (GCs), including individual ID as a random intercept, and collection date and food supplementation as fixed effects. GCs concentrations were log-transformed to improve model fit. Shannon Indices were Tukey transformed prior to analysis to achieve residual normality. All models were assessed for multicollinearity among predictor variables by calculating variance inflation factors (VIF < 5).

### Differential abundance testing

To identify the bacterial taxa whose relative abundances were significantly associated with changes in host GCs, we constructed negative binomial mixed models and implemented our analysis using the NBZIMM package^96^. Negative binomial models outperform other traditional differential abundance methods (e.g. DESeq) because they are better equipped to handle the zero-inflation and sparsity common to microbiome count data^96^. Taxa included in differential abundance testing were filtered with a liberal threshold of > 0.001% relative abundance to the overall microbiome community to avoid excluding rare taxa as they contribute substantially to measures of community diversity^58,97^. Models included the read count of each bacterial taxa as the dependent variable, GCs (scaled to zero mean and unit variance) as a fixed effect, controlling for collection date (fixed), food supplementation (fixed), and individual id (random). Taxa whose negative binomial models did not converge due to a high presence of zeroes were modeled instead with zero-inflated negative binomial models using the glmer.zinb() function in the same package (NBZIMM). We controlled the false discovery rate by applying a Benjamini–Hochberg FDR correction to all p-values. Adjusted p-values < 0.05 were considered statistically significant.

### Structural equation modelling

To integrate ecological and host variables into our model framework investigating the relationship between GCs and gut microbiome diversity, we constructed a structural equation model using (SEM) using the piecewiseSEM package^69^. SEM is an effective way to evaluate direct and indirect effects of multiple variables within complex ecological systems^43^. Unlike traditional variance covariance-based SEM, piecewise SEM approaches allow for the inclusion of random effects, the construction of a single causal network from multiple separate models, and the ability to handle small sample sizes and compare models using Akaike information criterion (AIC)^69^.

Using piecewiseSEM, we investigated whether the relationship between GCs and gut microbiome diversity (endogenous variables, i.e., variables of interest) was moderated by host and/or ecological factors (exogenous variables, i.e., variance outside of the model structure). All categorical variables were converted to numeric variables prior to modeling. To build the SEM, we first constructed two component linear mixed-effect models. The first model tested the effects of conspecific density, reproductive activity, dietary heterogeneity, and an upcoming mast on GCs. The second model tested the effects of reproductive activity, dietary heterogeneity, an upcoming mast, age, and GCs on gut microbiome diversity (Chao1 richness). Both component models included sample collection date and food supplementation status as a fixed effect and individual ID as a random effect. However, food supplementation did not affect GCs or gut microbiome diversity in either of the component models (effect on GCs: β ± SE − 4.86 ± 3.08, t = − 1.58, P = 0.12; effect on gut microbiome alpha diversity: β ± SE − 29.14 ± 79.13, t = − 0.37, P = 0.71), and was therefore removed from the SEM to improve model fit (AICc) and refine the standardized beta estimates. The overall fit of the SEM was evaluated using Shipley’s test of d-separation Fisher’s C statistic and AICc.

## Supplementary Information

Supplementary Information.

## Data Availability

All sequences, hormone data, and R code related to this manuscript are available at figshare (10.6084/m9.figshare.19077773 and https://figshare.com/s/a52886d8016cdd1f0dbb).
