# Supplementary material for: Glucocorticoids coordinate changes in gut microbiome composition in wild North American red squirrels
Source: Sci Rep. 2022 Feb 16;12:2605. doi: 10.1038/s41598-022-06359-5 (PMC8850573; doi:10.1038/s41598-022-06359-5)
Supplement: Supplementary file 1 — Supplementary Information. [file 41598_2022_6359_MOESM1_ESM.pdf]

Supplementary materials for:

Glucocorticoids coordinate changes in gut microbiome composition in wild North American red squirrels

Lauren Petrullo[1], Tiantian Ren[2], Martin Wu[2], Rudy Boonstra[3], Rupert Palme[4], Stan Boutin[5], Andrew G. McAdam[6], Ben Dantzer[1,7]

Figure S1. *A priori* structural equation model depicting hypothesized pathways and directionality among variables. Black solid lines represent hypothesized significant positive relationships; red solid lines represent hypothesized significant negative relationships.

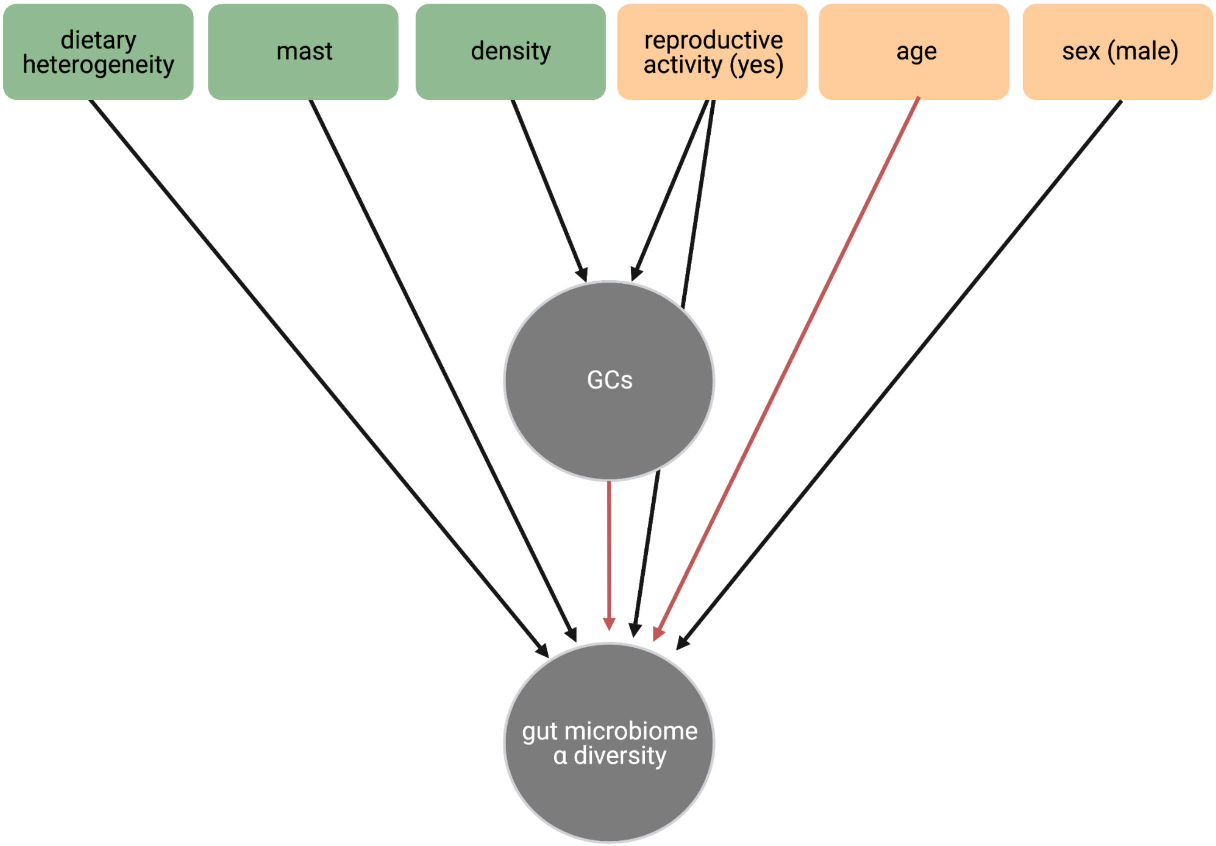

Table S1. Differentially abundant taxa at the family level. Significant (FDR-adjusted P value < 0.05) results from negative binomial mixed models testing the effect of GCs on each bacterial family controlling for collection date and food supplementation status.

bacterial-family	model-estimate	p-value	fdr-adjusted-p	change-with-GCs
[Odoribacteraceae]	-6.83	0.00	0.00	DECREASE
Sporichthyaceae	-3.17	0.00	0.00	DECREASE
Xanthomonadaceae	-1.39	0.00	0.00	DECREASE
Chitinophagaceae	-1.38	0.00	0.00	DECREASE
Christensenellaceae	-1.01	0.00	0.00	DECREASE
Bacillaceae	-0.84	0.00	0.01	DECREASE
Elusimicrobiaceae	-0.81	0.00	0.00	DECREASE
Flavobacteriaceae	-0.64	0.00	0.00	DECREASE
Staphylococcaceae	-0.64	0.01	0.05	DECREASE
Streptomycetaceae	-0.63	0.00	0.00	DECREASE
Sinobacteraceae	-0.57	0.00	0.00	DECREASE
Ruminococcaceae	0.12	0.00	0.02	INCREASE
Coriobacteriaceae	0.57	0.00	0.01	INCREASE
Streptococcaceae	1.00	0.00	0.00	INCREASE
Dermabacteraceae	2.81	0.00	0.00	INCREASE

Table S2. Differentially abundant taxa at the genus level. Significant (FDR-adjusted P value < 0.05) results from negative binomial mixed models testing the effect of GCs on each bacterial genus controlling for collection date and food supplementation status.

bacterial-genus	model-estimate	p-value	fdr-adjusted-p	change-with-GCs
Kribbella	-650.76	0.00	0.00	DECREASE
Salmonella	-10.98	0.00	0.00	DECREASE
Odoribacter	-6.83	0.00	0.00	DECREASE
Skermanella	-2.85	0.00	0.00	DECREASE
Acidisoma	-2.35	0.00	0.00	DECREASE
Stenotrophomonas	-2.29	0.00	0.00	DECREASE
Rahnella	-2.17	0.00	0.00	DECREASE
Dyadobacter	-1.68	0.00	0.00	DECREASE
Actinomycetospora	-1.49	0.00	0.00	DECREASE
Variovorax	-1.42	0.00	0.00	DECREASE
Pseudoclavibacter	-1.24	0.00	0.00	DECREASE
Modestobacter	-1.19	0.00	0.00	DECREASE
Brevundimonas	-1.17	0.01	0.02	DECREASE
Yersinia	-1.08	0.00	0.00	DECREASE
Hyphomicrobium	-1.00	0.00	0.01	DECREASE
Reyranella	-0.88	0.00	0.00	DECREASE
Roseomonas	-0.87	0.00	0.00	DECREASE
Bacillus	-0.77	0.00	0.02	DECREASE
Brochothrix	-0.76	0.00	0.00	DECREASE
Flavobacterium	-0.64	0.00	0.00	DECREASE
Steroidobacter	-0.57	0.00	0.00	DECREASE
Ochrobactrum	-0.35	0.01	0.04	DECREASE
Clostridium	0.28	0.00	0.01	INCREASE
Butyricicoccus	0.35	0.00	0.00	INCREASE
Kurthia	0.46	0.00	0.00	INCREASE
Microbacterium	0.52	0.01	0.04	INCREASE
Oscillospira	0.60	0.00	0.00	INCREASE
YRC22	0.65	0.00	0.00	INCREASE
Lachnospira	0.67	0.00	0.02	INCREASE
Pedomicrobium	0.96	0.00	0.00	INCREASE
Streptococcus	1.00	0.00	0.00	INCREASE
Actinomyces	1.01	0.00	0.00	INCREASE
Eggerthella	1.15	0.00	0.00	INCREASE
Ralstonia	1.38	0.00	0.00	INCREASE
Brachybacterium	2.81	0.00	0.00	INCREASE
Janthinobacterium	2.82	0.00	0.00	INCREASE
Propionibacterium	274.64	0.00	0.00	INCREASE
